# Hypoxia Abrogates Tumor-Suppressive Activities of C/EBPδ in Pancreatic Cancer

**DOI:** 10.3390/ijms25179449

**Published:** 2024-08-30

**Authors:** Leonie Hartl, Marieke S. ten Brink, Hella L. Aberson, Jan Koster, Danny A. Zwijnenburg, JanWillem Duitman, Maarten F. Bijlsma, C. Arnold Spek

**Affiliations:** 1 Center for Experimental and Molecular Medicine, Laboratory for Experimental Oncology and Radiobiology, Amsterdam UMC Location University of Amsterdam, 1105 AZ Amsterdam, The Netherlands; 2 Cancer Center Amsterdam, Cancer Biology and Immunology, 1081 HV Amsterdam, The Netherlands; 3 Center for Experimental and Molecular Medicine, Division of Infectious Diseases, Amsterdam UMC Location University of Amsterdam, 1105 AZ Amsterdam, The Netherlands; 4 Department of Pulmonary Medicine, Amsterdam UMC Location University of Amsterdam, 1105 AZ Amsterdam, The Netherlands; 5 Department of Experimental Immunology, Amsterdam UMC Location University of Amsterdam, 1105 AZ Amsterdam, The Netherlands; 6 Amsterdam Infection & Immunity, Inflammatory Diseases, 1105 AZ Amsterdam, The Netherlands

**Keywords:** CEBPD, hypoxia, HIF1A, pancreatic cancer

## Abstract

Pancreatic ductal adenocarcinoma (PDAC) is a dismal disease with a low 5-year survival rate of only 13%. Despite intense research efforts, PDAC remains insufficiently understood. In part, this is attributed to opposing effects of key players being unraveled, including the stroma but also molecules that act in a context-dependent manner. One such molecule is the transcription factor C/EBPδ, where we recently showed that C/EBPδ exerts tumor-suppressive effects in PDAC cells in vitro. To better understand the role of C/EBPδ in different contexts and the development of PDAC, we here build on these findings and assess the effect of C/EBPδ in a PDAC model in mice. We establish that the lack of oxygen in vivo—hypoxia—counteracts the tumor-suppressive effects of C/EBPδ, and identify a reciprocal feedback loop between C/EBPδ and HIF-1α. RNA sequencing of C/EBPδ-induced cells under hypoxia also suggests that the growth-limiting effects of C/EBPδ decrease with oxygen tension. Consequently, in vitro proliferation assays reveal that the tumor-suppressive activities of C/EBPδ are abrogated due to hypoxia. This study demonstrates the importance of considering major physiological parameters in preclinical approaches.

## 1. Introduction

Pancreatic ductal adenocarcinoma (PDAC) is among the deadliest cancers of all. Due to a late onset of symptoms, PDAC is typically diagnosed at an advanced stage, rendering 80–85% of patients ineligible for surgical resection [1]. Treatment options for these patients remain poor, resulting in a 5-year survival rate of only 3% in patients with distant metastases [2]. The incidence of PDAC keeps rising by about 1% every year, resulting in an estimated 66,440 new cases and 51,750 deaths from pancreatic cancer in the US in 2024, and making it the third leading cause of cancer-related death [2]. It is therefore of utmost importance that we better understand the biology of PDAC and utilize state-of-the-art models to study its molecular characteristics and improve patient treatment.

CCAAT/enhancer-binding protein delta (C/EBPδ) belongs to the C/EBP-family of transcription factors which is associated with the development of various malignancies [3]. Depending on the biological context, C/EBPδ has been shown to function as a tumor suppressor or as a tumor promoter, employing various mechanisms [3,4,5]. While C/EBPδ can induce differentiation in cancer cells thereby limiting their proliferation, C/EBPδ has also been shown to act as a mediator of cancer cell stemness and epithelial-to-mesenchymal transition (EMT) [6,7,8,9]. Furthermore, C/EBPδ can aid in hypoxia adaption, boost angiogenesis, and modulate inflammatory signaling to promote tumor growth [10,11,12,13]. The biological context and specifically the interactions between tumor cells and the stromal compartment thus appear to contribute to the outcome of C/EBPδ’s activity [4].

We previously showed that C/EBPδ protein and mRNA are suppressed in PDAC tumor cells and that lower C/EBPδ expression correlates with enhanced lymph node involvement and shorter overall survival [14]. Re-expression of C/EBPδ in different PDAC cell lines curbed proliferation, clonogenicity, and migration, suggesting it has predominantly tumor suppressive activities in this context [14,15]. These experiments were performed using classical 2-dimensional cell culture models. While being valuable models for early-stage preclinical experiments, these 2-dimensional models lack many characteristics of the physiological tumor microenvironment like a 3-dimensional extracellular matrix, interstitial pressure, interactions with stromal cells, and a restricted access to nutrients. In this study, we consequently sought to confirm the effects of C/EBPδ on PDAC development and test whether re-expression of C/EBPδ also limits tumor growth in a pre-clinical subcutaneous in vivo model and under hypoxic conditions.

## 2. Results

### 2.1. C/EBPδ Limits PDAC Cell Expansion without Exerting Paracrine Effects

To test whether C/EBPδ functions as a tumor suppressor not only in vitro but also in vivo, a co-injection model of inducible C/EBPδ overexpressing and wild type PDAC cells was developed. The inducibility of C/EBPδ using doxycycline in these cells was previously confirmed on an mRNA and protein level [14], and Figure S1 exemplarily shows that the known C/EBPδ target genes *IL8* and *PTX3* [16,17] are also induced upon C/EBPδ activation, implying that functional C/EBPδ is made in response to doxycycline treatment.

**Figure 1:**
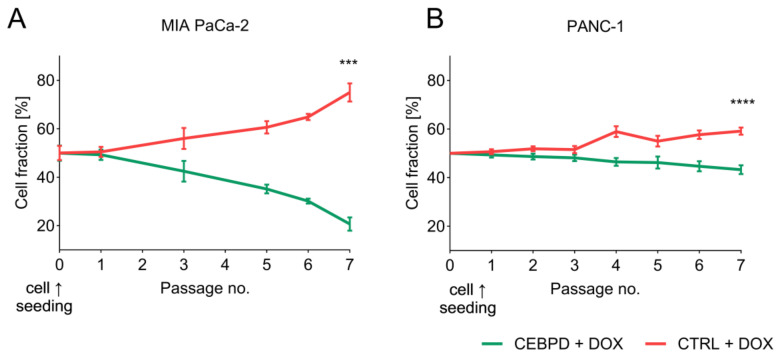
Competition assay of *CEBPD*-inducible and control (CTRL) cells. (**A**) MIA PaCa-2 or (**B**) PANC-1 cells harboring a doxycycline-inducible overexpression construct for *CEBPD* expression (*CEBPD*, green lines) or a control construct (CTRL, red lines) were mixed at equal ratios (cell seeding, passage 0) and treated with doxycycline (DOX). Cells were cultured for a period of 4 weeks with two passages per week and the ratio of both daughter cell lines—CEBPD and CTRL—was determined at each passage using fluorescence activated cell sorting (FACS). These data are corrected for cell line-specific differences in proliferation observed in untreated cells and normalized to t = 0. Experiments were conducted in triplicate, shown as the mean ± SEM. *** *p* < 0.001; **** *p* < 0.0001.

To exclude possible paracrine effects, we first tested the effect of C/EBPδ on (neighboring) tumor cells in vitro using a doxycycline-inducible *CEBPD* overexpression system [14]. Specifically, we initiated a competition assay using doxycycline-inducible PDAC cells, where inducible cells are green fluorescent and control cells are red fluorescent. Inducible and control cells were combined, treated with doxycycline, and followed over the course of several passages. To control for clonal differences in proliferation, we conducted the same experiment without doxycycline and corrected the expansion rate of the treated cells for the difference observed in untreated cells (Figure S2). From flow cytometry analysis, it became clear that in the doxycycline-treated condition, the fraction of inducible, C/EBPδ-high cells decreased while the fraction of control, C/EBPδ-low cells increased after normalization to the untreated control condition (Figure 1). These data confirm the previously established notion that C/EBPδ reduces the proliferative potential of PDAC cells, presumably via intracellular mechanisms, whilst the growth of neighboring cells remains unaffected. Why the difference in proliferative potential is amplified in MIA PaCa-2 as opposed to PANC-1 remains unknown, but it is conceivable that the higher growth rate of MIA PaCa-2 and the different methylation profiles of the two cell lines account at least in part for this [18].

### 2.2. Growth-Limiting Effects of C/EBPδ Are Diminished In Vivo

**Figure 2:**
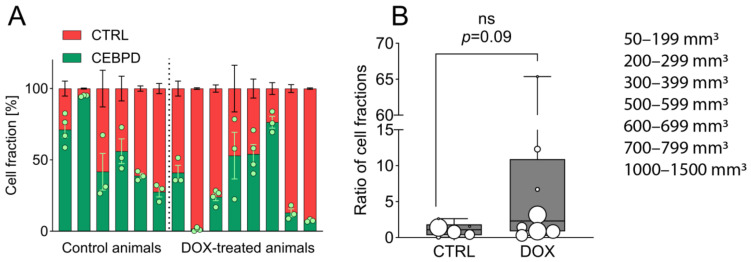
Subcutaneous competition assay of C/EBPδ-inducible and CTRL cells. (**A**) FACS analysis was used to determine the fraction of inducible (green) and CTRL (red) MIA PaCa-2 cells in subcutaneous tumors of doxycycline-treated and untreated mice (n = 3 or 4 samples per mouse/tumor). Green dots represent individual tumor samples. Red and green cell counts were normalized to those in the control group and normalized counts were transposed to 100% per mouse. Shown is the mean ± SEM. (**B**) Shown is the ratio of green and red fractions as derived from (**A**) (ratio = red fraction/green fraction). The size of marks correlate with tumor volumes at the day of sacrifice. One-tailed Mann–Whitney U test shows no significant difference in ratio between treated (DOX) and untreated (CTRL) tumors (*p* = 0.09). ns = not significant.

After ruling out C/EBPδ-mediated paracrine effects on proliferation in vitro, we next tested the effect of C/EBPδ on PDAC cell proliferation in vivo. To this end, we employed a subcutaneous xenograft mouse model using MIA PaCa-2 cells where a combination of inducible and control cells were injected in the flank of immune-deficient nude mice. Mice were then treated with or without doxycycline for up to 8 weeks. From analyzing the ratio of green fluorescent inducible and red fluorescent control cells in harvested tumors, it became apparent that C/EBPδ induction in cancer cells in vivo did not show as distinct growth-suppressive effects as it did in vitro (Figure 2A). Plotting the ratio of red control and green inducible cell fractions suggested that control cells in the doxycycline-treated group only had a minor growth advantage over C/EBPδ-induced cells and that these ratios do not significantly differ between the two treatment arms (Figure 2B). Although the lack of significance might be attributed to low sample sizes and enhanced biological variability in vivo as compared to in vitro, these outcomes suggest that the downstream effects of C/EBPδ differ between in vitro and in vivo conditions. While C/EBPδ activation in vitro resulted in a marked growth advantage of control cells over C/EBPδ-induced cells after 4 weeks, we merely observed a trend reminiscent of this in vivo. Interestingly, although doxycycline treatment did slightly favor the outgrowth of control cells over C/EBPδ-inducible cells, doxycycline-treated tumors were on average larger than untreated ones (52%, 708 mm^3^, and 464 mm^3^, respectively). Altogether, these results imply that the proliferation-limiting effects of C/EBPδ induction observed in vitro are diminished through yet unknown mechanisms in vivo.

### 2.3. C/EBPδ Is Expressed near Necrotic, HIF-1α-Positive Areas in Subcutaneous Tumors

**Figure 3:**
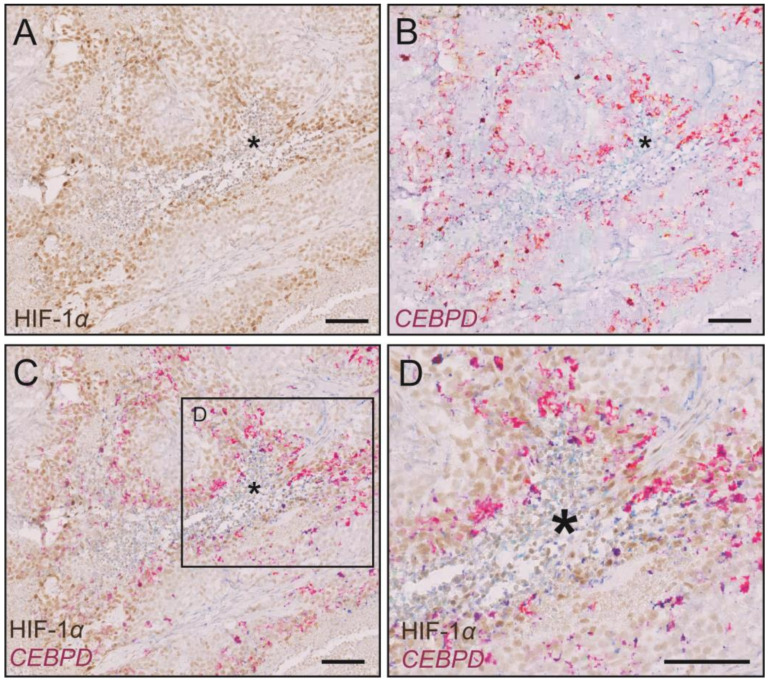
HIF-1α protein and *CEBPD* mRNA in PDAC xenografts. (**A**) HIF-1α protein staining, (**B**) *CEBPD* mRNA staining, and (**C**,**D**) overlay of HIF-1α protein and *CEBPD* mRNA staining. Scale bar is 100 µm. * marks an area of cells undergoing uncontrolled cell death due to insufficient levels of oxygen (necrotic area).

Multiple parameters differ between the in vitro setting and the in vivo environment. To some extent, these differences could potentially be attributed to the absence of stromal cells in the in vitro experiments. Indeed, pancreatic cancer is known for its very dense desmoplastic reaction, for high interstitial pressure, and low oxygen tension [19]. To obtain insights into the mechanisms underlying the lack of effects of C/EBPδ on proliferation in vivo, we first used fluorescence microscopy to look at cross-sections of the tumors and further found that red control cells preferably grow at the periphery of the tumors while C/EBPδ-inducible cells also proliferate at the core of the tumor mass (Figure S3). This suggests that cells expressing C/EBPδ can better cope with the harsh environment at the center of a tumor. The tumor core is indeed characterized by increased interstitial pressure and by decreased oxygen tension. To corroborate these findings, we used RNA in situ hybridization to visualize *CEBPD* mRNA in sections of the obtained xenografts (Figure 3B–D). We opted for RNA in situ hybridization for *CEBPD* to directly assess the in vivo efficacy of doxycycline which acts on *CEBPD* transcription and because we previously showed that high levels of *CEBPD* mRNA correspond to high levels of C/EBPδ protein in our C/EBPδ overexpressing MIAPaCa-2 cells. From these, it became clear that *CEBPD* is predominantly expressed in the periphery of necrotic areas. Necrotic areas, also referred to as necrotic cores, are masses of cells undergoing a form of uncontrolled cell death (necrosis) in response to insufficient levels of oxygen. Cells undergoing necrosis disintegrate and release their contents and debris into their surroundings, further triggering a local inflammatory response. C/EBPδ has previously been shown to be expressed in the (still) viable cells immediately surrounding necrotic areas where it is co-expressed with HIF-1α, a major transcriptional regulator of the cellular response to hypoxia [11]. We thus next stained HIF-1α protein in consecutive sections (Figure 3A,C,D). Importantly, as opposed to *CEBPD*, HIF-1α must be assessed on the protein level as its mRNA is highly abundant while the protein is rapidly degraded under oxygenated conditions, therefore serving as the more reliable readout of HIF-1α activity. These stainings revealed that *CEBPD* is indeed highly expressed in the cells surrounding the necrotic, hypoxic, HIF-1α-positive areas which are filled with collapsed cells (marked with asterisks in Figure 3). While HIF-1α is widely expressed in these tumors, quantitative image analysis shows that around 70% of the *CEBPD*-associated signal overlaps with the HIF-1α protein signal. In summary, these findings imply that C/EBPδ-inducible cells can better cope with the harsh environment at the center of tumors than the non-C/EBPδ-inducible control cells.

### 2.4. C/EBPδ Is Induced by Hypoxia and Boosts HIF-1a Protein Expression

**Figure 4:**
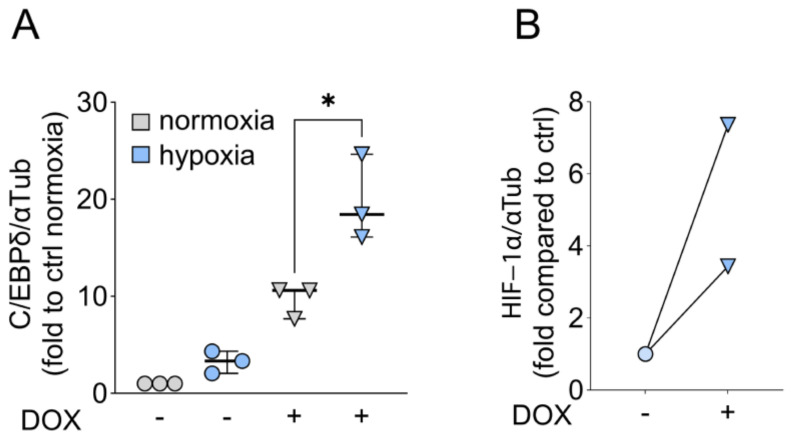
Hypoxia induces C/EBPδ in PDAC cell lines. (**A**) C/EBPδ protein is induced in MIA PaCa-2 cells upon doxycycline treatment (DOX, half-filled circles) as opposed to untreated cells and even more so in the presence of hypoxia (blue bars, n = 3). One-tailed Mann–Whitney test shows that C/EBPδ induction is significantly improved under hypoxia (* *p* = 0.05). Data are normalized to C/EBPδ expression in untreated cells under normoxia. (**B**) HIF-1α expression in MIA PaCa-2 cells before and after induction of C/EBPδ under hypoxic conditions (n = 3). Data are normalized to HIF-1α levels in untreated cells.

Cells that are cultured in vitro are commonly exposed to 20% oxygen (normoxia). However, most tumors—and especially PDAC which presents with a dense desmoplastic reaction—have to cope with lower oxygen tensions of 0.3–4.2% (hypoxia) which can have important consequences for tumor cell behavior [20]. This, and the results in Figure 3, prompted us to further elucidate the role of C/EBPδ in PDAC cells under oxygen-deprived conditions. The effect of hypoxia on the cells was confirmed by HIF-1α protein expression which is elevated in hypoxic cells compared to normoxic cultures where HIF-1α protein was barely detected regardless of C/EBPδ induction (Figures S4 and S5). This assured us that the cells sense the lack of oxygen and exhibit the appropriate response (Figure S6). Further, C/EBPδ was induced by hypoxia as compared to normoxia, irrespective of the addition of doxycycline (Figure 4A). Also, doxycycline-mediated induction of C/EBPδ was much more potent under hypoxia than under normoxic oxygen levels (Figure 4A). As a feedback mechanism has been described between C/EBPδ and HIF-1α, we next looked at HIF-1α protein levels in C/EBPδ-induced cells. We indeed observed that HIF-1α expression is reciprocally enhanced when C/EBPδ was induced (Figure 4B). Likewise, we have observed that *HIF1A* mRNA levels are increased upon the induction of C/EBPδ under both normoxic and hypoxic conditions (Figure S7). Interestingly, however, it has been shown in alveolar epithelial cells that acute hypoxia induces HIF-1α protein stability, while prolonged hypoxia of 12 h leads to a decrease in both *HIF1A* mRNA and HIF-1α protein levels [21]. Based on these data and our observation that hypoxia initially induces C/EBPδ, it is conceivable that acute hypoxia jump-starts a sustainable and prolonged feedback reaction between C/EBPδ and HIF-1α.

In conclusion, we found that hypoxia promotes a favorable environment for C/EBPδ activation and that high expression of HIF-1α correlates with high levels of *CEBPD* mRNA and protein expression.

### 2.5. Hypoxia Prevents C/EBPδ-Mediated Suppression of Proliferation-Associated Pathways

**Figure 5:**
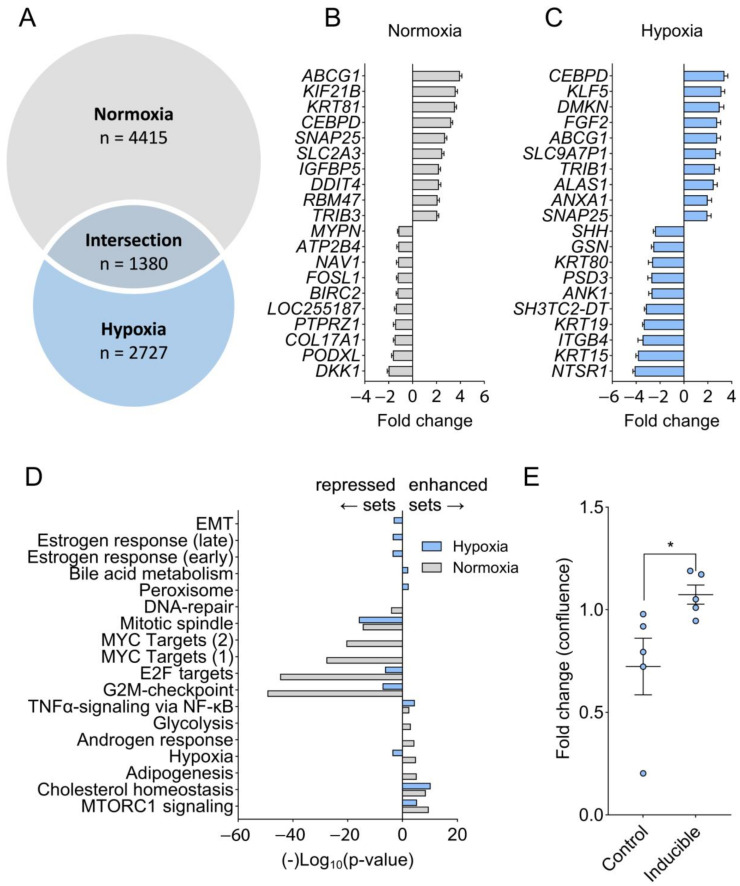
RNA sequencing analysis of C/EBPδ’s downstream effects under normoxia and hypoxia. (**A**) Venn diagram showing differentially expressed genes (DEGs) before and after C/EBPδ induction under hypoxia (blue) and normoxia (gray) for 48 h and the overlapping fraction. Depicted is the number of genes contained in each fraction. Experiments were run using biological triplicates, DEGs were determined using DESeq2. (**B**,**C**) Bar graphs displaying the 10 most up and downregulated genes—of the 100 most significantly changed ones—upon induction of C/EBPδ under normoxia (**B**) and hypoxia (**C**). (**D**) All DEGs after C/EBPδ induction under normoxia (gray bars) and hypoxia (blue bars) were correlated with 50 computationally generated gene sets describing distinct biological states (Molecular Signatures Database Hallmark gene sets [22]) using the build-in gene set analysis function in the Genomics Analysis and Visualization Platform R2 [23]. −Log10 of the significance of overlap is plotted on the y-axis whereby enriched datasets are plotted above y = 0 and depleted sets are plotted below y = 0 (using Log10 (*p*-value) instead of the negative Log10). EMT = epithelial-to-mesenchymal transition. (**E**) Proliferation under hypoxia is negatively affected by doxycycline in control cells (Control) but promoted in C/EBPδ-inducible cells (Inducible). Confluency is normalized to that measured at t = 0 and fold changes are calculated for both conditions as (confluency [doxycycline]/confluency [untreated] at 72 h post-treatment). * *p* = 0.0159, 2-sided Mann–Whitney U test, shown as the mean ± SEM.

To obtain a mechanistic insight into why C/EBPδ limits tumor growth in normoxia but does this less efficiently in hypoxia, we subjected MIAPaCa-2 control cells and C/EBPδ-inducible cells at hypoxia and at normoxia to C/EBPδ induction and to subsequent RNA sequencing. Figure 5A shows a Venn diagram of the differentially expressed genes (DEGs) due to C/EBPδ induction for 48 h under both conditions. The top 10 up and downregulated genes are listed in Figure 5B (normoxia) and Figure 5C (hypoxia). We next determined the enrichment of the DEGs in both conditions within the Hallmark gene sets (Broad institute Molecular Signatures Database [22]) (Figure 5D). Interestingly, proliferation-associated MYC targets are suppressed by C/EBPδ under normoxia but not under hypoxia. Cell cycle progression is hampered by C/EBPδ under normoxia through suppression of the G2M checkpoint and of E2F targets, while under hypoxia, suppression of these gene sets is much less pronounced. It thus seems that multiple mechanisms are differentially regulated by C/EBPδ under hypoxia and the limited downregulation of cell cycle progression and proliferation likely contributes to the lack of C/EBPδ’s tumor-suppressive effects under hypoxic conditions. Indeed, we have previously shown that induction of C/EBPδ in these cells reduced their proliferation under normoxic conditions (Figure 1A and [14], while here, we present a cell growth assay under hypoxic conditions, showing that doxycycline-induced C/EBPδ even leads to a slight increase in proliferation in the absence of oxygen (Figure 5E).

## 3. Discussion

In this study, we challenged the role of C/EBPδ as a tumor suppressor in PDAC using a preclinical mouse model. While we have previously reported a growth-suppressive effect of C/EBPδ under normoxic conditions in vitro, we here show that in hypoxia and in vivo, these tumor suppressor effects are abrogated, presumably due to reciprocal activation of C/EBPδ and HIF-1α. Mechanistically, RNA sequencing suggests that under normoxic conditions, C/EBPδ strongly suppresses MYC-mediated proliferation as well as the G2M checkpoint and E2F-mediated cell cycle progression. This suppression is weakened (E2F targets and G2M checkpoint) or absent (MYC targets) under hypoxia, presumably contributing to a growth advantage of C/EBPδ-high cells under hypoxia as opposed to its proliferation-limiting effects previously observed under normoxia [14].

The current literature indeed points towards a positive feedback loop of C/EBPδ and HIF-1α, a major regulator of the cellular response to hypoxia and predictor of poor prognosis in PDAC [4,24,25,26,27]. In breast cancer cells, C/EBPδ is induced by hypoxia [10]. In response to this, C/EBPδ suppresses the ubiquitin ligase FBXW7 which otherwise targets mTOR for proteasomal degradation. Suppression of FBXW7 consequently reactivates mTOR/AKT/S6K1 signaling which subsequently activates HIF-1α [10]. Additionally, C/EBPδ promotes HIF-1α expression through upregulation of the IL-6 receptor in these cells, enhancing their self-renewal and sphere-forming capacity [8]. Suppression of HIF-1α on the other hand led to inhibition of C/EBPδ in lymph endothelial cells, implying a regulatory mechanism in the opposite direction [24]. In macrophages, C/EBPδ aids in HIF-1α-mediated inflammatory signaling and macrophage activation [28]. Although the latter two have not been assessed in tumor cells, these findings point to a reciprocal action between the two transcription factors. Our findings add to this notion; we found an increase in *HIF1A* mRNA due to C/EBPδ induction (Figure S7) and also HIF-1α protein appears to be induced by C/EBPδ expression. At the same time, C/EBPδ is robustly induced by hypoxia. These data confirm the described reciprocal loop, and further complicate the notion of C/EBPδ as a tumor suppressor. Interrupting the HIF-1α/C/EBPδ axis might yet present a clinically interesting route to exploit the tumor-suppressive effects of C/EBPδ in hypoxic PDAC tumors.

HIF-1α is a subunit of the HIF-1 transcription factor and possesses transcriptional activity. Whether HIF-1α directly interferes with C/EBPδ-mediated transcription, or whether HIF-1α-mediated transcription counteracts that by C/EBPδ remains unclear. The latter would imply that under severe hypoxia (i.e., <1% oxygen), HIF-1α mediated transcription might outperform C/EBPδ-mediated suppression.

In apparent contradiction to the findings presented above, we previously showed that C/EBPδ expression in PDAC tumor cells—known to be particularly a hypoxic tumor—positively correlates with patient survival [14]. Conversely, here we found that re-expression of C/EBPδ in hypoxic tumor cells cannot compensate for the effects of low endogenous C/EBPδ expression under hypoxia. C/EBPδ is known to be involved in various tumor-related mechanisms including inflammatory and endothelial cell signaling. Appropriate tumor–stroma interactions are absent in xenograft models where human tumor cells are nested in a mouse stroma. It might thus be the crosstalk between tumor cell-C/EBPδ and stromal cells that ultimately determines patient survival. Alternatively, human PDACs might be less hypoxic than these largely avascular subcutaneous tumors, causing only a diminished influence on C/EBPδ’s downstream effects. Next to this, we showed a negative correlation of C/EBPδ and lymph node involvement in PDAC patients. Lymph node involvement can function as a predictor of distant metastases which are the main reason for cancer-related death [29]. In agreement with this, we have previously shown that C/EBPδ is a major regulator of cell cytoskeleton dynamics in PDAC cells and that cells with higher C/EBPδ have a lower propensity to migrate [15]. Importantly, HIF-1α has recently been shown to act as a suppressor of cell motility and metastasis in orthotropic PDAC mouse models [30]. Although this is in discrepancy with other findings [25,31], it makes us question HIF-1α’s reputation of a general marker for poor prognosis. Whether an induction of C/EBPδ contributes to this effect remains to be elucidated. Yet, while aiding cell survival in hypoxic tumor regions, C/EBPδ might still limit the dissociation of cells from the primary tumor through cytoskeletal retardation and thereby hamper metastases formation and enhance patient survival.

A positive feedback loop has also been described between C/EBPδ and MYC which co-amplify in urothelial carcinoma (UC) to promote a metabolic shift from oxidative phosphorylation towards glycolysis [32]. In UC cells, this relationship is—again—in part regulated by C/EBPδ-mediated repression of FBXW7 to prevent proteasomal degradation of MYC [32]. HIF-1α on the other hand has been shown to suppress MYC via multiple pathways to reduce proliferation [33,34]. Whether HIF-1α-mediated repression of MYC is ineffective in PDAC cells or counteracted by C/EBPδ-mediated activation of MYC remains unclear. Yet, conversely, HIF-1α carefully controls residual MYC-activity to promote angiogenesis and glycolysis to ultimately enhance cell survival [33,34]. Both C/EBPδ and HIF-1α might thus contribute to the reduced suppression of MYC targets upon C/EBPδ induction under hypoxia and promote tumor cell survival in vivo.

Unfortunately, mouse models do not perfectly resemble human cancers and neither does a hypoxia chamber. Hypoxic monoculture of tumor cell lines still only resembles a small part of the intricate physiology of tumors. Like hypoxia, the presence of immune cell signaling and other stroma-dependent parameters can distort the effects of C/EBPδ. Currently, improved in vitro models are being developed which more closely resemble the in vivo situation including a low oxygen tension, high interstitial pressure, and the presence of stromal cells [35]. Such models could reduce the use of animals in pre-clinical research and enhance clinical translation efficacy, leading to more efficient use of funding.

## 4. Materials and Methods

### 4.1. Cell Lines and Cell Culture

MIA PaCa-2 (RRID: CVCL_0428, CRL-1420, ATCC, Manassas, VA, USA), PANC-1 (RRID: CVCL_0480, CRL-1469, ATCC), and Hs 766T (RRID: CVCL_0334, HTB-134, ATCC, Manassas, VA, USA) cells were maintained in DMEM medium (#41965120, Gibco, Waltham, MA, USA) supplemented with 9% (*v*/*v*) fetal bovine serum (FBS) (#S-FBS-NL_015, Serana, Pessin, Germany), 2% (*v*/*v*) penicillin–streptomycin (#15140122, Gibco), and 2 mM L-glutamine (#17-605E, Lonza, Basel, Switzerland), hereafter referred to as complete growth medium, at subconfluency in a tissue culture incubator in 5% CO_2_ at 37 °C or in a hypoxia chamber at 1% oxygen and 37 °C. Cells were monthly tested negative for mycoplasma and their identity was confirmed yearly by STR profiling.

### 4.2. Cloning Strategy and Lentiviral Transduction

C/EBPδ-inducible and Control (CTRL) cells were generated as described previously [14]. In short, MIA PaCa-2 (CRL-1420, ATCC) and PANC-1 cells (CRL-1469, ATCC) were transduced with the pCW57 vector (#80921, Addgene, Watertown, MA, USA) containing the CEBPD cDNA (C/EBPδ-inducible cells) or with the empty vector (CTRL cells). To distinguish C/EBPδ-inducible cells from CTRL cells, green and red fluorophores were added through lentiviral transduction as described before [15].

### 4.3. Competition Assay

Control and doxycycline-inducible cells (5 × 10^4^ per cell line) were mixed and seeded in 6-well plates after which they were treated with doxycycline at a concentration of 2 µg/mL to induce C/EBPδ expression or left untreated. Twice per week, cells were detached using TripLE Express Enzyme (#12604013, Gibco), washed in phosphate-buffered saline (PBS), and resuspended in FACS-buffer (1% FBS in PBS). To determine cell fractions, fluorescence-activated cell sorting was performed on the CytoFLEX S Flow Cytometer (Beckman Coulter, Brea, CA, USA). Cell fractions were quantified using the CytExpert Acquisition and Analysis Software Version 2.4. Instrument settings were calibrated before each use using the CytoFLEX Daily QC Fluorospheres (#B53230, Beckman Coulter). Briefly, single cells were computationally separated from doublets and debris and the fraction of red fluorescent (ECD-A channel) and green fluorescent (FITC-A channel) cells were calculated. Graphs were made using GraphPad Prism (version 9.1.0, GraphPad Software Inc., San Diego, CA, USA).

### 4.4. Animal Experiments and Declarations

All animal experiments were approved by the Animal Experimentation Committee of the Academic Medical Center Amsterdam and conducted in accordance with national guidelines. Ethical approval was granted by the Central Animal Experiments Committee (Centrale Commissie Dierproeven). Age-matched female athymic nude mice were purchased from Envigo and housed on a 12 h light-dark cycle at 20–26 °C. Animals were randomly assigned to experimental groups prior to tumor cell injection. Before tumor cell injections, animals were anaesthetized in 1.5–2% isoflurane in 100% oxygen. Doxycycline-inducible and control MIA PaCa-2 cells (1 × 10^6^ cells in 50% Matrigel (#356231, Corning, Glendale, AZ, USA)) were mixed at equal ratios and subcutaneously injected into the flank of 8 mice per treatment group. Mice in the treatment group received 2 g/L doxycycline in the drinking water supplemented with 1% glucose (#G8769, Merck, Amsterdam, The Netherlands) which was refreshed 2–3 times per week. Tumors were measured twice per week in three directions and the volume was calculated as (length × width × height)/2. Upon reaching a tumor volume of 1 cm^3^, or a humane endpoint, mice were sacrificed by cervical dislocation under anesthesia with 4–5% isoflurane in 100% oxygen. Harvested tumors were parted and either stored in 0.9% NaCl for single-cell dissociation and FACS analysis or in 10% neutral buffered formalin (NBF) for sectioning.

### 4.5. Single Cell Dissociation and FACS Analysis

Tumors were washed in ice cold PBS, minced using a scalpel on ice and subsequently processed as essentially described before [36]. Briefly, minced tumors were incubated for 60 min a 37 °C with a dissociation enzyme cocktail containing 1 mg/mL Type VIII Collagenase (#C2139, Sigma-Aldrich, Saint Louis, MO, USA), 2 mg/mL Dispase II (#4942078001, Sigma-Aldrich), 1 mg/mL Soybean Trypsin Inhibitor (#17075029, Gibco), and 1 unit/mL DNase I (#M0303S, New England Biolabs, Ipswich, MA, USA) dissolved in PBS. Per tumor, 4 samples were collected and diluted in 10 mL of complete growth medium to inactivate dissociating enzymes, passed through a 70 µm cell strainer, pelleted, and re-suspended in FACS buffer for FACS analysis as described in Section 2.3.

### 4.6. Fluorescence Analysis of Tumors

For cryosections, tissues were fixed in 10% neutral buffered formalin (NBF) overnight and then saturated in 30% sucrose overnight. The Cryostat CryoStar NX70 (Epredia, Breda, The Netherlands) was used to slice 10 µm thick sections which were mounted using ProLong Gold Antifade Mountant (#P36930, Thermo Fisher Scientific, Waltham, MA, USA) on SuperFrost specimen holders (Epredia). Sections were imaged using the EVOS FL Cell Imaging System (Thermo Fisher Scientific).

### 4.7. Immunohistochemistry

For immunohistochemistry, samples were fixed in 10% NBF overnight and dehydrated through an ethanol series before embedding them in paraffin. Sections 5 µm thick were deparaffinized and re-hydrated followed by antigen retrieval in citrate buffer at pH 6. Endogenous peroxidase was blocked using Dako REAL Peroxidase-Blocking Solution (#S2023, Agilent, Santa Clara, CA, USA), slides were rinsed in PBS, and blocked using Ultra V Block (#TA-125-UB, Thermo Scientific) for 10 min at room temperature. Primary antibodies (1:500; rabbit-anti-HIF-1α, #36169, Cell signaling, Danvers, MA, USA; and 1:200; α-SMA, #ab5694, Abcam, Cambridge, UK) were diluted in antibody diluent (#UD09-500, Immunologic) and incubated overnight at 4 °C. The next day, slides were rinsed in PBS, incubated with BrightVision one component detection system anti-rabbit IgG HRP (#DPVR-55HRP, Immunologic) for 30 min followed by incubation with 3,3′-Diaminobenzidine (BS04-999, Immunologic) for 1 min, 2 rinses in demineralized water and hematoxylin-counterstaining for 1 min (#VWRK4085.9001, Avantor, Amsterdam, The Netherlands). Sections were rinsed in tab water, cleared in xylene, and mounted using Pertex (#00811-EX, Histolab, Askim, Sweden).

### 4.8. RNAscope

To visualize *CEBPD* mRNA in consecutive sections, the RNAscope system (RNAscope 2.5 HD Assay—Brown, #322300, Advanced Cell Diagnostics, Newark, CA, USA) was used according to the supplier’s protocol; slides were pre-heated to 60 °C, deparaffinized, twice incubated in 100% ethanol for 1 min, and air-dried. Endogenous peroxidase was quenched using the RNAscope Hydrogen Peroxide (#322330) for 10 min at room temperature. Heat-induced antigen retrieval was performed at 99 °C using 1× RNAscope Target Retrieval Reagent (#322000) for 15 min, followed by a wash in demineralized water and incubation in 100% ethanol for 3 min. Protease treatment was performed at 40 °C using the RNAscope Protease Plus Reagent (#322330) for 30 min. The undiluted target probe (Probe-Hs-CEBPD-O1, #831431), positive control probe (PPIB, #300031), and negative control probe (dapB, #310043) were applied to different sections and incubated for 2 h at 40 °C. Slides were washed in 1× wash buffer (#310091) and incubated with amplification probes 1, 2, 3, and 4 (Amp 1/2/3/4, #322310) for 30, 15, 30, and 15 min with intermittent washes in 1 wash buffer. Amp 5/6 were incubated for 30 and 15 min at room temperature with intermittent washes. Sections were incubated with RNAscope diaminobenzidine (DAB, #322300) for 10 min at room temperature, washed, counter-stained with Mayer’s Hematoxylin (#VWRK4085.9001, Avantor) diluted 1:2 in demineralized water for 2 min, and rinsed under tap-water. Stained slides were dehydrated in an ethanol series followed by xylene and mounted using Pertex (#00811-EX, Histolab, Askim, Sweden). All slides were scanned using the Philips IntelliSite Ultra Fast 1.6 slide scanner.

### 4.9. Protein Analysis Using Capillary Western Immunoassay (WES)

Cells (1 × 10^5^) were seeded in 24-well plates under the respective experimental conditions. Upon treatment, cells were lyzed using 4x Laemmli sample buffer (#1610747, Bio-Rad, Veenendaal, The Netherlands) supplemented with 2% β-mercaptoethanol on ice and boiled for 10 min. HIF-1α and C/EBPδ protein levels were subsequently quantified using the WES Simple Western capillary-based automated immunoassay system (ProteinSimple, San Jose, CA, USA) according to the supplier’s protocol. In short, 2 µL protein lysate mixed with fluorescent master mix, blocking reagent, primary antibodies (anti-C/EBPδ antibody;1:20; sc-365546, Santa Cruz Biotechnology, Dallas, TX, USA; anti-HIF-1α antibody; 1:20; #36169, Cell Signaling; and anti-α-Tubulin; 1:50; #sc-2398, Santa Cruz Biotechnology), HRP-conjugated secondary antibodies, and chemiluminescent substrate was pipetted into the WES detection module. Next, the plate was run using default instrument settings consisting of: stacking and separation at 375 V for 25 min, blocking reagent for 5 min, primary and secondary antibody both for 30 min. The obtained electropherograms were finally processed and analyzed using the Compass software (ProteinSimple, software version 5.0.1) after which the obtained chemiluminescence signal of the protein of interest (C/EBPδ or HIF1α) was divided by that of the house keeping protein (α-Tubulin).

### 4.10. RNA Extraction and RNA Sequencing

Cells were seeded in 12-well plates (3.5 × 10^5^ per well), either treated with doxycycline at 2 µg/mL to induce C/EBPδ expression or left untreated and incubated for 48 h under normoxia or hypoxia. Cells were then lysed and RNA was extracted and processed as described before [15]. Sequencing libraries were prepared and sequenced using the Illumina NovaSeq 6000 (Illumina, San Diego, CA, USA) as described before [15].

### 4.11. Proliferation Assay

Control and doxycycline-inducible cells were seeded at increasing confluences (1.25 × 10^3^–2 × 10^4^ per well) in 96-well plates. After cells attached, 2 µg/mL doxycycline were added to half of the wells in each condition and plates were transferred to hypoxia (1% oxygen) for 72 h. Plates were imaged using the IncuCyte S3 Life-Cell Analysis System (Satorius, Göttingen, Germany) at t = 0 h and at t = 72 h after doxycycline treatment. The IncuCyte Life Cell Analysis Software version 2021C was used to quantify cell confluence and the doxycycline-induced fold change was calculated as (confluence [doxycycline-treated]/confluence[untreated]) after correcting for differences in confluence at t = 0.

### 4.12. Bioinformatics Analyses

RNA sequencing of doxycycline-induced and control cells under normoxia has been described and published previously (Gene Expression Omnibus, accession number GSE214028) [15]. RNA sequencing of induced and control cells under hypoxia (for 48 h) was performed and analyzed similarly and can be found at Gene Expression Omnibus under accession number GSE226038. Briefly, the RNA sequence read quality was assessed using FastQC (version 0.11.5). Reads were aligned to the human reference genome (NCBI37/hg19) using STAR v2.7.1 and annotated with Gencode v32. The R2 Genomics Analysis and Visualization Platform [23] was used to determine differentially expressed genes using the DESeq2 package (version 1.18.1) with a cutoff at FDR-corrected *p* < 0.01. The same platform was used to conduct gene set analyses involving the Broad 2020 Hallmark gene set collection [22].

### 4.13. Quantitative Image Analysis

The images shown in Figure 3A,B were used to correlate the localization of *CEBPD* mRNA and HIF-1α protein signals in tumors. Quantitative image analysis was performed using ImageJ Version 1.53 [37] and the JACoP plugin [38]. Images were prepared in Adobe Photoshop Software (version 23.4.2) by converting them to 16-bit depth, selecting the magenta channel, and inverting the gray values. Automatic thresholding was used to calculate the Overlap Coefficient and Mater’s Coefficients (the fraction of each respective signal overlapping with the other signal).

### 4.14. Statistical Analyses

All statistical tests, sample sizes, and error bar definitions are given in the respective figure legend. The *t*-tests and Mann–Whitney U tests were conducted in GraphPad Prism (version 9.1.0, GraphPad Software Inc.), and differential gene expression analysis and gene set analysis were performed using the Genomics Analysis and Visualization Platform R2 (R2.amc.nl, accessed on 15 June 2022) [23]. All graphs were made in GraphPad Prism (version 9.1.0, GraphPad Software Inc.).

## 5. Conclusions

Although different in vitro models have shown a growth-suppressive effect of C/EBPδ in PDAC cells, we were unable to show a significant C/EBPδ-induced reduction of proliferation of these cells in vivo and under hypoxic in vitro conditions. Instead, we point out a reciprocal loop of C/EBPδ and HIF-1α, the main mediator of the response to hypoxia, which likely contributes to the loss of C/EBPδ’s tumor suppressive effect. Our findings emphasize the fact that C/EBPδ acts context-dependently and urges the use of improved pre-clinical models to enhance successful clinical translation.

## Data Availability

RNA sequencing of doxycycline-induced and control-cells under normoxia has been described and published previously (Gene Expression Omnibus, accession number GSE214028) [15]. RNA sequencing of induced and control cells under hypoxia can be found at Gene Expression Omnibus under accession number GSE226038.

## Supplementary Materials

The following supporting information can be downloaded at: https://www.mdpi.com/article/10.3390/ijms25179449/s1.
