# Supplementary material for: Hypoxia Abrogates Tumor-Suppressive Activities of C/EBPδ in Pancreatic Cancer
Source: Int J Mol Sci. 2024 Aug 30;25(17):9449. doi: 10.3390/ijms25179449 (PMC11394991; doi:10.3390/ijms25179449)
Supplement: Supplementary file 1 [file ijms-25-09449-s001.zip › ijms-3155934-supplementary.pdf]

Supplementary Materials Data

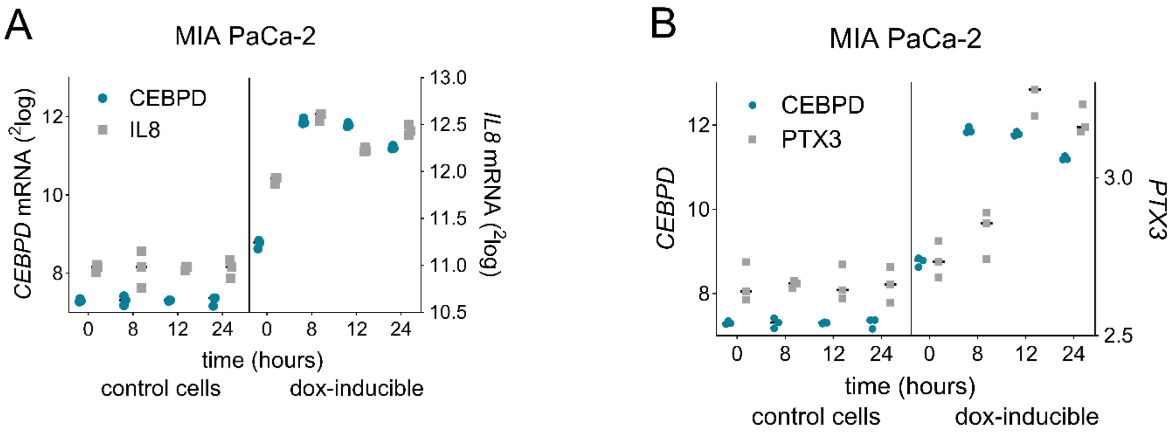

Figure S1. Target genes of C/EBPδ are induced upon Dox-induction. mRNA expression of the C/EBPδ target genes *IL8* (**A**) and *PTX3* (**B**) are correlated over time with the mRNA expression of *CEBPD* in doxycycline-treated MIA PaCa-2 cells transduced with an empty control plasmid (control cells) and in MIA PaCa-2 cells transduced with a doxycycline-inducible construct for *CEBPD* over-expression.

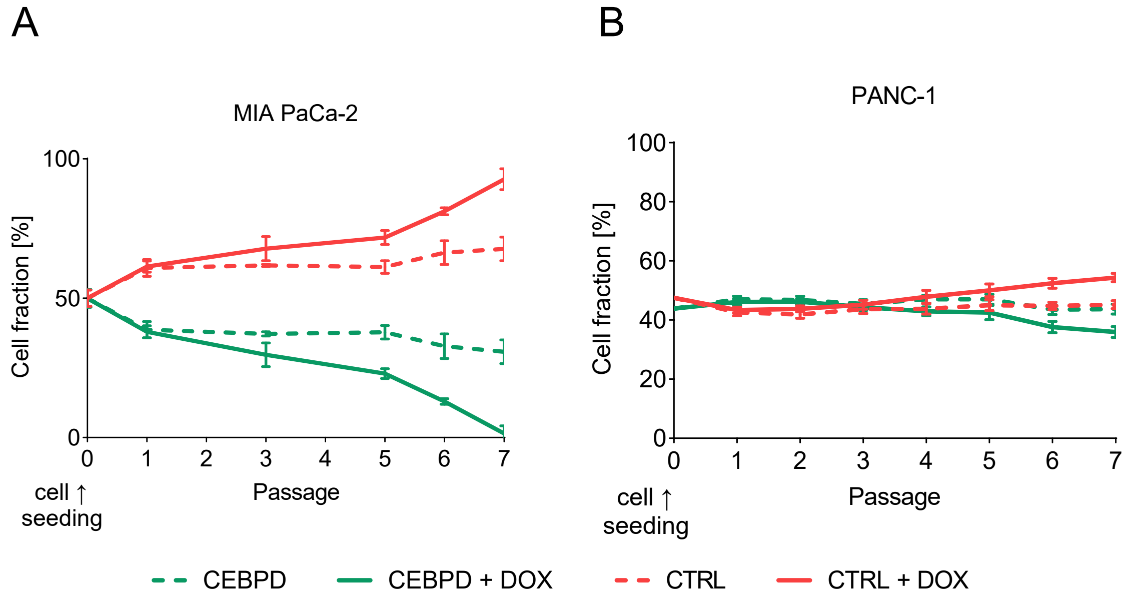

Figure S2. Raw data of competition assay. MIA PaCa-2 (**A**) and PANC-1 (**B**) cells transduces with either an empty control plasmid (red lines) or a doxycycline-inducible plasmid for CEBPD over-expression (green lines) were either treated with doxycycline (full lines) or left untreated (dotted lines) and the ratio of the control and inducible cells was followed over time. To correct for clonal differences, the data were normalized to the profile of the control cells.

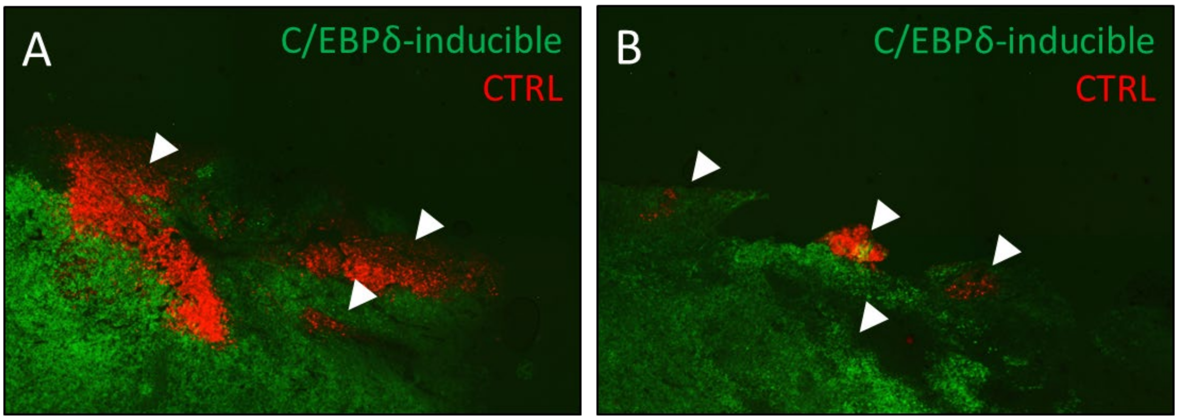

Figure S3. Fluorescent images of sectioned subcutaneous tumors. Red fluorescent cells (control cells, white arrow heads) preferably grow at the periphery of the tumor while green (C/EBPδ-inducible) cells comprise the core of the tumor. (**A**) is derived from a mouse treated with doxycycline, (**B**) from an untreated control animal.

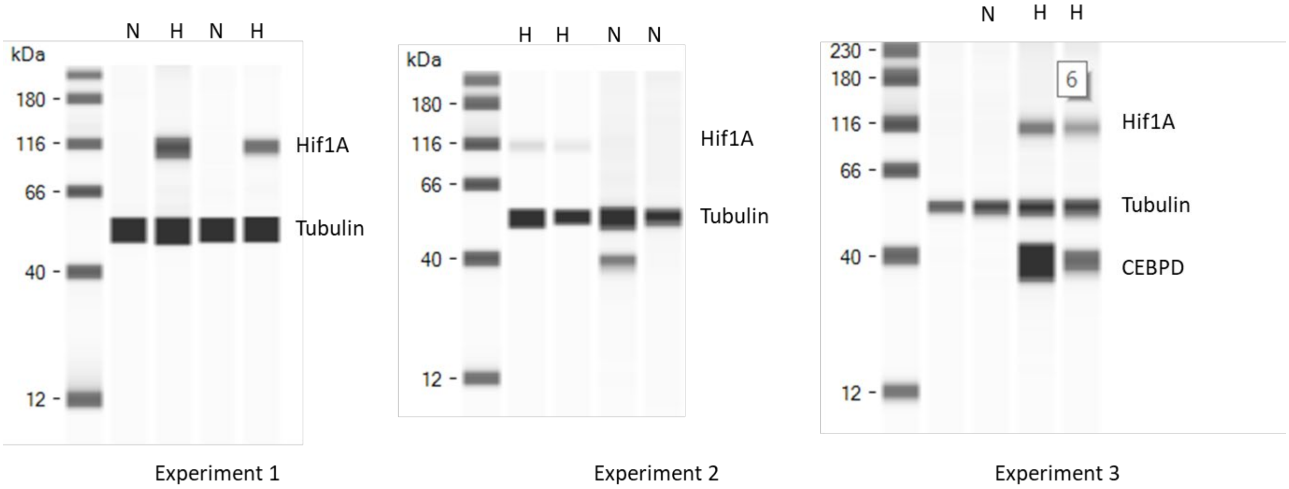

Figure S4. Simple Western blot lane view for data shown in Figure S6 (i.e., Normoxia (N) versus Hypoxia (H)).

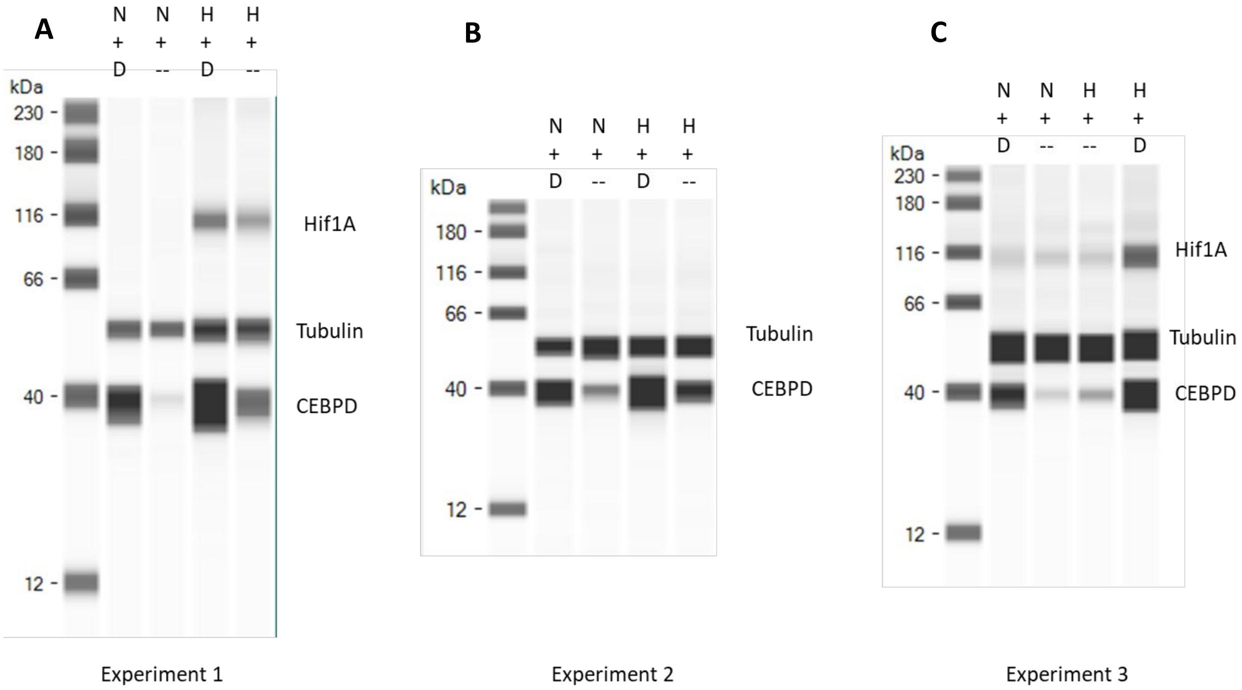

Figure S5. Simple Western blot lane view for data shown in Figure 4A (panels (**A**–**C**); i.e. Normoxia (N) or Hypoxia (H) in combination with DOX (D) or control (--) stimulation) and Figure 4B (panels (**A**,**B**); i.e., Hypoxia (H) in combination with DOX (D) or control (--) stimulation).

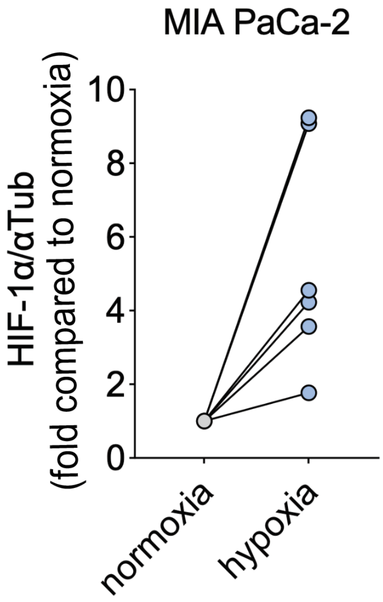

Figure S6. Induction of HIF-1α protein in MIA PaCa-2 cells under hypoxia.

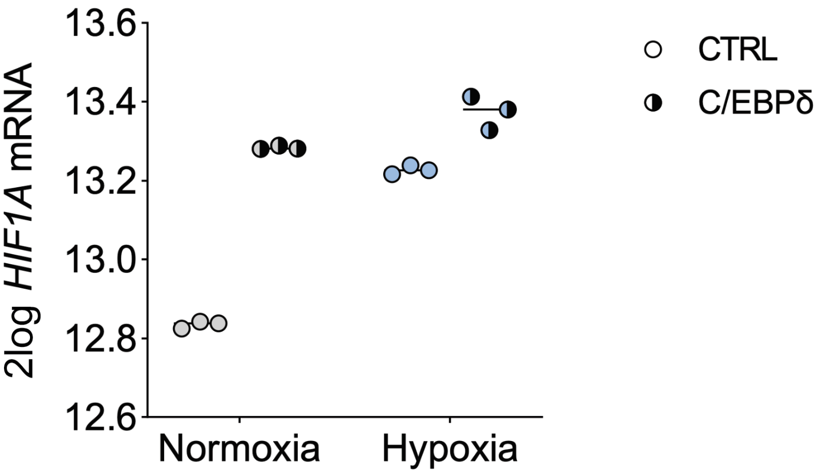

Figure S7. *HIF1A* mRNA expression is enhanced upon C/EBPδ induction in MIA PaCa-2 cells under normoxia and further enhanced under hypoxia. Data are derived from RNAseq data at available at Gene Expression Omnibus under accession number GSE226038.
